# Uncertainty visualization of gaze estimation to support operator-controlled calibration

**DOI:** 10.16910/jemr.10.5.6

**Published:** 2018-01-25

**Authors:** Almoctar Hassoumi, Vsevolod Peysakhovich, Christophe Hurter

**Affiliations:** École Nationale de l’Aviation Civile , France; ISAE-SUPAEROFrance

**Keywords:** eye movement, eye tracking, uncertainty, head movement, smooth pursuit, accuracy, gaze estimation, accuracy improvement, usability

## Abstract

In this paper, we investigate how visualization assets can support the qualitative evaluation of gaze estimation uncertainty. Although eye tracking data are commonly available, little has been done to visually investigate the uncertainty of recorded gaze information. This paper tries to fill this gap by using innovative uncertainty computation and visualization. Given a gaze processing pipeline, we estimate the location of this gaze position in the world camera. To do so we developed our own gaze data processing which give us access to every stage of the data transformation and thus the uncertainty computation. To validate our gaze estimation pipeline, we designed an experiment with 12 participants and showed that the correction methods we proposed reduced the Mean Angular Error by about 1.32 cm, aggregating all 12 participants' results. The Mean Angular Error is 0.25° (SD=0.15°) after correction of the estimated gaze. Next, to support the qualitative assessment of this data, we provide a map which codes the actual uncertainty in the user point of view.

## Introduction

While eye tracking data are ubiquitous with various activities like human-computer interaction assessment and user behaviour analysis, little has been done to visually investigate the uncertainty of recorded gaze information. One of the main reasons for this gap is the complexity of gaze processing, starting from pupil detection up to gaze location in the user’s field of view (i.e. world space or world camera space). Gaze processing is considered to be a system with pupil center detection as entry raw data and the user gaze location as the output data. This paper tries to fill this gap by providing evidence that gaze processing can be depicted considering the uncertainty assessment. Furthermore, we provide an innovative visualization of uncertainty map computation which is based on the standard heat map where the kernel size is adjusted by the pupil location and its corresponding uncertainty.

Estimating user gaze with high accuracy and good precision has long been the utmost objective in HumanComputer Interaction and eye tracking research. Binocular eye trackers offer a good level of accuracy but involve supplementary materials and are expensive. The objective of this paper is to advance research in monocular eyetracking research and help to make calibration procedure endeavors to be more precise, accurate yet succinct. It is crucial to investigate approaches to reduce calibration errors instead of restarting the calibration procedure. While there are plenty of empirical studies of calibration procedures, relatively limited progress has been made toward correcting estimated gaze error, reducing the time required, or making the calibration less tedious. Hence, we achieve more natural eye tracking calibration with the following benefits: 1) the calibration procedure is easier to perform, 2) gaze estimation is more precise and accurate, resulting in a Mean Angular Error of 0.25° (SD 0.15°) after applying the error correction methods we propose and 3) uncertainty is visually inspected.

The remainder of the paper is as follows: First, we explain our gaze processing pipeline. This processing uses a standard head-mounted eye tracking system, where we capture the pupil video stream and the world camera. We then explain our calibration method and clarify its intrinsic calibration uncertainty. A method to correct the estimated gaze positions is presented afterward. Next, we explain the global data processing uncertainty propagation. To provide further insight into our method, we illustrate our findings with two application use cases where the calibration uncertainty is shown with recorded user gaze data. Finally, we discuss our findings and outline potential further studies.

## Related Work

### Related works on pupil detection algorithms:

Robust gaze tracking is strongly related to accurate eye features detection. The most salient elements in the eye image are the sclera – the white outer layer of the eyeball –, the iris – the contractile muscle forming the colored portion of the eye – and the pupil – the contracting aperture through which light enters the eye. It is surprising to find that there is a wide variety of pupil detection methods that are developed for the same purpose, that is, to detect the true center locations[ 9 ], in some cases, the contour of the pupil area. In video-based oculography, visible or infrared imaging data are used. The latter uses either bright pupil or dark pupil images[ 18 ],.Kondou and Ebisawa[ 15 ], used near-infrared LEDs arranged around each of the stereo cameras and configured them so that they were able to turn on and off synchronously. This way, the authors could obtain consecutive bright pupil and dark pupil images. Then, they computed the difference between the two images, making pupil detection easier. Some pupil detection techniques employ a histogram based threshold [ 11 ] and give relatively good results under laboratory conditions. For example, in Starburst [ 16 ], an adaptive threshold was used on a region of interest to localize corneal reflection, then the corneal reflection was removed from the image using radial interpolation. Thereafter, the pupil contour candidates were detected using rays coming from the best guess of the pupil center. 

In 2012, an algorithm employing coarse positioning using Haar-like features was proposed by Swirski et al. [ 26 ]and, through their self-designed open source eye tracking system, Pupil Labs [ 14 ], they exhibited an approach in which edges were detected using a Canny filter. Darker areas are then searched from lowest spike in histogram and pupil candidates are obtained using ellipse fitting.

More recently, Fuhl presented ExCuSe: an algorithm based on morphologic operations and the Angular Integral Projection Function to detect pupil contour [ 8 ]. However, they still seem to face the same challenges. The algorithms tend to be less robust in real-world environments and changing light conditions, occlusions, viewing angles and head poses. To address those issues, ElSe [ 9 ], analogous to ExCuSe, used Canny edge filter and if no ellipse was found, a second analysis was conducted. It first estimates a likely candidate and then refines its position. The results of a recent experiment designed by Fuhl et al [ 9 ]. showed that the ElSe algorithm offers the best results in terms of accurate pupil positions on challenging everyday images when compared with state-of-the-art pupil detection algorithms. However, the methods exhibited above do not mention the uncertainty of their pupil center detection. It is most likely that different pupil detection methods yield different pupil center locations using the same eye image, even if they are slightly different. In this paper, we address the crux of this issue by investigating an area that the pupil center is likely to be in and we propose a method to visually inspect this area.

### Related works on gaze estimation:

Based on the information retrieved from the eye image, the gaze is estimated. There are two different leading approaches for estimating gaze position: feature– based and appearance-based gaze estimation. Because of its design and geometric-based aspect, the latter does not ask for a calibration routine, however, the feature-based approach requires eye image informative characteristics, namely the pupil center position and, in some cases, the corneal reflections as an input to provide the gaze position. This approach again splits into two different methods: model-based and interpolation-based approaches, banking on the type of mapping used to calculate the gaze output from the pupil feature(s) input. In most model-based approaches, the eye is modeled in 3D and gaze vector direction is calculated [ 24 ]. Interpolation-based approaches are the most recently used method in both remote and obtrusive eye tracking systems. The method calls for the use of mapping functions which are based on neural networks or polynomial regressions [5].

In the case of parametric functions, the parameters are computed during a calibration routine. Polynomial regression has gained considerable interest in recent studies. Cerrolaza et al. compared over 400,000 forms of parametric mapping functions [5]. Many polynomial expressions with different orders have been tested by Blignaud [1], Mitsugami et al. [17] and Cerrolaza et al. [5] used a second order polynomial in x and y with first order combinations. Furthermore, nonparametric functions enable the pupil features to be mapped to the point of regard by means of a trained neural network.

### Toward the calibration data recording pattern:

The community’s standard and most used eye tracking calibration pattern is the 9-point visual stimulus calibration. Nonetheless, whereas the aim is to present the points sequentially to cover a large part of the visual scene, Pfeuffer et al. [20] described it as tedious, dull and tiring for the eyes. Recent studies [22] showed that, unlike the conventional fixed-point calibration procedures, the approaches based on moving targets tend to be faster and reliable. Typically, the most convenient feature of this approach is the ability to obtain a larger amount of unique pupil-marker center tuples at various scene areas. In their Pursuit Calibration method, Pfeuffer et al. investigated a smooth pursuit calibration where they considered one moving marker at a constant velocity following a rectangular path. Similarly, Celebi et al. [3] used smooth pursuit for their calibration technique, however, in contrast with Pursuit Calibration, a more predictable path followed by the marker was presented in their paper. Namely, they used an Archimedean spiral trajectory with constant linear velocity (6.4°/sec), circumventing the problems raised by the path used in Pursuit Calibration [20]: Following only the border of the rectangle may not help to retrieve the interior points and the rectangle’s corners may induce instabilities due to the abrupt change in the trajectory direction. However, to alleviate the corner instability problem, Pfeuffer et al. designed an experiment where they considered a constant speed target trajectory and an accelerated speed target trajectory where the target moved slowly, close to the corners, enabling a more natural transition [20].

Celebi et al. applied quadratic regression to find the mapping function that is used to produce gaze estimation from the eye position [3]. They corrected the lag between the smooth pursuit motion and the actual target positions and then they discarded the outliers using a simple fit residual rejection criterion applied three times. The results showed that the RMS of the non-truncated data of the smooth pursuit calibration was 0.838° (SD=.278, 27 seconds calibration time) compared to the 9-point calibration which gave 1.388° (SD=.963, 23 seconds calibration time). The authors truncated the smooth pursuit calibration data in order to consider a similar time to the 9-point calibration for proper comparison and obtained an error of 0.913° (SD=0.272). Pfeuffer’s method builds on the correlation between the eye movement and the target’s trajectory using Pearson’s product-moment in a userdefined moving window. The mapping model is obtained using the homography computation of OpenCV with RANSAC for outlier removal. The authors reported an accuracy under 1° for both the constant and accelerated speed calibration greater than 10 s. However, they did not apply estimation corrections to their results.

In Evans et al. [7], the authors investigated the collection of calibration points while following a supervisor’s thumb relocated to five different positions, compared with an approach consisting of a user looking at a fixed point and moving his head in an asterisk-like trajectory. Approximately 20 calibration points were gathered, and an offline calibration computation gave a mean error of 0.83°. Similarly, in CalibMe [22], the authors proposed a method to collect a large array of calibration points using automatically detecting fiducial markers, without the assistance of a supervisor. They also came up with a custom outliers’ removal method. Also, they provided a parameterizable method to automatically reserve evaluation points. Because the calibration procedure enabled a large number of points to be assembled, evaluation points could be selected from among those collected points and the remaining points served to calculate the mapping function.

In their second calibration methods, Kondou and Ebisawa proposed a method in which one visual marker was shown on the screen and moved from a position P1 to a position P2 onward and backward [15]. The user was asked to fixate on the marker during the entire movement, making a smooth pursuit eye movement. The results showed that the moving target calibration was better than the two-fixedpoints calibration also proposed in the same paper. Significant differences between the two calibration methods (P<0.05) using a t-test were found. Unfortunately, no information about the Mean Angular Error was provided by the authors. Also, although their methods seem to give good results, supplementary materials were used (stereo wide view cameras and an additional pan-tilt-zoom camera) and NIR LEDs arranged around each camera.

## Gaze Estimation Method

In this section, we explain the gaze data processing. The gaze positions are estimated using pupil center positions and a mapping function obtained during a calibration procedure.

### Overview of the Gaze Estimation System

**Figure 1:**
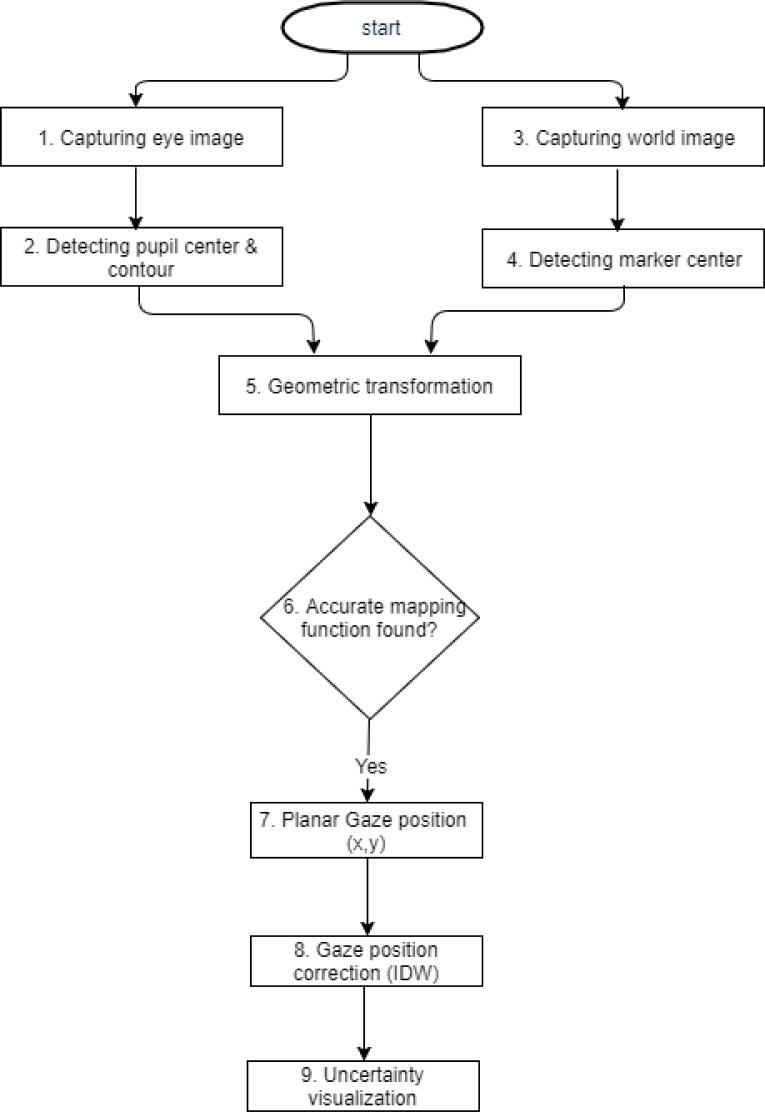
Flowchart of the gaze estimation method.

Figure 1 presents an overall view of the gaze estimation system developed. Initially, the pupil center of the subject was detected and tracked with a Pupil Lab eye tracking system’s eye Camera. We used the device equipped with one eye camera and a world camera. Detailed explanations of the pupil center detection are given in the following subsection Pupil Center Detection. A custom marker present in the large field-of-view of the Pupil Lab World Camera was detected, as explained in subsection Marker Detection (step 3 and 4 of Figure 1). This marker will serve later on for the calibration. After that, the subject performed one of the two following calibration procedures: fixating on the center of the marker while moving his head to make a rotation as in CalibMe [22] or fixating on a moving object while keeping his head still as in Pursuit calibration [20].

**Figure 2:**
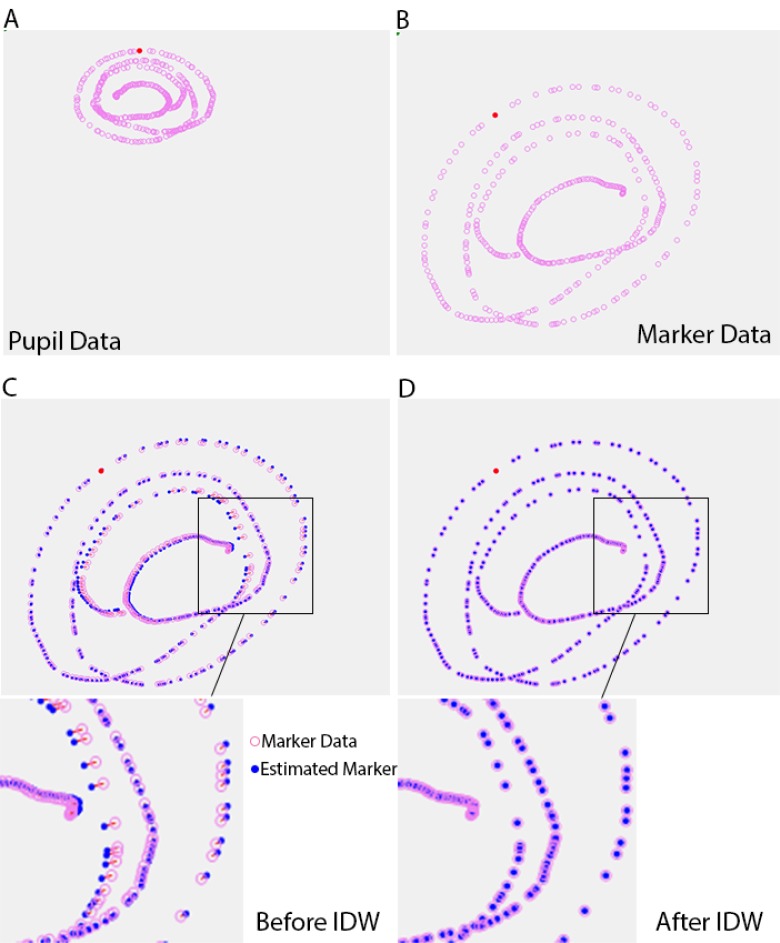
the user interface of ELAN. The software supports multiple synchronized media sources and an arbitrary number of annotation tiers. Videos are blurred to protect participants.

In a pilot study, we asked some participants to do a different calibration procedure which consisted of fixating on a moving target (smooth pursuit) and rotating their head (vestibule-ocular movement) at the same time. The participants reported that this calibration procedure was difficult as they found it arduous and uncomfortable. Thus, we removed it from our experiment setup. After the initial stage of the calibration procedure, the pupil and the marker centers’ coordinates were gathered and stored for further processing. Each pupil center position corresponds to a marker center position at a specific time. Thus, the same number of pupil centers and marker centers are stored as pairs.

The two sets of gathered data are used to get coefficients of a mapping function using a bivariate second order polynomial regression. This mapping function will be used to calculate the final planar gaze estimations based on new pupil centers given as input. After obtaining the mapping functions, we can then estimate the marker positions with the pupil center positions obtained during the calibration procedure to verify the reliability of the function. The estimated positions do not have exactly the same positions as the actual marker positions. We then correct the positions of those estimated marker centers with Inverse Distance Weighting [25]. Thus, every pupil center detected next will be corrected by Inverse Distance Weighting. Finally, we propose a method based on kernel density to visualize the uncertainty of the overall gaze estimation.

### Pupil Detection (Center + Area)

 Accurate pupil center detection [9] is the first and most important step of accurate gaze estimation. This section corresponds to step 2 of Figure 1. While there are many pupil detection algorithms that proved to give good results [9, 13, 14], and they would probably perform well in this study, we have developed our own easy and fast detection algorithm for flexibility and to have full control of the processes which will help with the visualization of the uncertainty (Uncertainty Computation Section). The pupil detection algorithm implemented in this study locates the features of the dark pupil present in the IR illuminated eye camera frame. Since our paper focuses on uncertainty visualization from pupil detection, the calibration algorithm is implemented so that the user can move his head freely, thus, we do not use pupil corneal reflection to compensate for small head movements. As such, we use the distance transform presented in [23] to compute the resulting uncertainty of the pupil area, taking into account the detected pupil center location. The outcomes of the algorithm were sufficient to obtain an accurate pupil area and center, and give valid results in laboratory conditions.

### Marker Detection

During the initial stage of user calibration, a user is asked to look at a reference point represented by the center of a marker. Choosing a marker to use is a well-studied problem [23]. A simple marker, the shape of which is not confused with any other object in the room, is appropriate; the marker must not have many details, so as to not distract the participant, and its center must easily be computable with affordable computer vision techniques. The marker consists of a thick black circle containing a white circle which in turn encompass a smaller filled black circle drawn on white paper, comparable to the markers used by Tobii and Pupil Labs. A white cross is drawn on its center. The marker is tracked using computer vision methods with OpenCv 3.1.

### Pairing Target and Pupil Center Positions

As the marker is fixed in a plane, the planar position of its center (Mx, My), obtained from the world camera, changes as soon as the subject moves his head or whenever the marker moves. In the same vein, if the subject is asked to look at the center (Mx, My) of the marker placed in the experimental environment while moving his head, the position of his pupil center (X_Pupil-center_, Y_Pupil-center_) changes accordingly. Namely, either gazing at the marker center while rotating the head, or fixating on the center of the marker while it moves, enables different paired positions of the marker and pupil centers to be obtained. Each marker center position then corresponds to a pupil center position. Thus, using the paired positions (pupil centersmarker centers) obtained, we can estimate gaze position using polynomial regression. The aim is to determine a transformation T such that the estimated gaze positions map as closely as possible to where the user is actually gazing. The result of this transformation is a form of isomorphism obtained thanks to a linear algebra method called Singular Value Decomposition SVD [4]. There are downsides of using a higher order polynomial in a visual stimuli-type calibration, and the quality can decrease if there are not enough points [1] .In most calibration procedures, a marker is used as visual stimuli. Five, nine or fifteen visual stimuli are displayed to the user. In our study, as the marker is not fixed in the world camera image, its position changes when the subject moves his head. The more the user moves and rotates his head in every direction, the more marker center positions are obtained and so the better the calibration process quality. For a set of n points, a polynomial of n order of less can be used. Consequently, care should be taken because going to a higher degree does not necessarily improve accuracy. The given known points may be accurate and well estimated by the polynomial regression, but the interpolated points may give surprisingly false results.

### Calibration Correction

Many feature-based calibration studies focused on comparing mapping functions [5] to pin down the best gaze estimation results. However, improving the results, reducing the error of gaze estimation after the monocular calibration, and minimizing the residuals have not been rigorously examined. In this section, by considering two different approaches, accurate methods to reduce calibration errors are proposed.

### Raw Estimated Gaze

We refer to raw estimated gazes as the gaze positions directly inferred by the mapping functions using pupil center locations as input.

### Inverse Distance Weighting

Inverse Distance Weighting (IDW) is an interpolation method that enables the unknown value of a point to be estimated according to the known values of its surrounding points based on their relative distance [25]. The theory is that nearby points devote more to the interpolated value than distant ones. The benefit of this method is that it is fast and easy to implement. The applicability of this method in this study is as follows: following the calibration procedure, paired pupil center and marker center points are gathered as shown in Figure 2 (A) and (B) respectively. Then, the same pupil center points are used to compute the estimated marker centers. Those estimated marker centers are called the reprojection points. They are slightly different from the actual marker centers. We now have a set of residuals which are the differences between the marker centers and the reprojection points.

Thereafter, a new pupil center will be used to estimate the gaze position and the error of this estimated gaze position with regard to the actual gaze position can be corrected because we know the errors of the surrounding points obtained during the calibration procedure. The closest points will contribute more to the correction and the furthest ones will have a small impact. The function of the IDW is given by:

(1) $$v_{p}=\frac{\sum_{i=1}^{n}v_{i}\frac{1}{d^{r}}}{\sum_{i=1}^{n}\frac{1}{d^{r}}}$$

Where v_j_ denotes the set of all correction vectors between the calibration points and the estimated points. d is the Euclidean distance between the calibration points P (x_j_,y_j_) and the estimated points P (x_p_ , y_p_ ) given by:

(2) $$d=\sqrt{{\left(x_{i}-x_{p}\right)}^{2}+{\left(y_{i}-y_{p}\right)}^{2}}$$

And r is a positive real number chosen arbitrarily. For this study, it was determined that the most appropriate value for r was 2.

### Modified Inverse Distance Weighting

The Modified Inverse Distance Weighting is original from this study. In this approach, the same function in (Eq.1) is applied. However, the calibration points used to approximate the interpolated value are selected differently. (Eq.1) is extended with the equation below:

(3) $${v_{s}=\forall e\in v_{i},|t}_{Np(x,y)}-t_{e}|\leq T$$

Where v_j_ is the set of all correction vectors between the calibration points and the estimated point, t_Np(x,y)_ is the timestamp of the nearest point, t_e_ is the timestamp of an element of the calibration points and T is the length of the time window which serves to verify if e is in the current set.v_s_ is the set of selected vectors contained in the current set.

**Figure 3:**
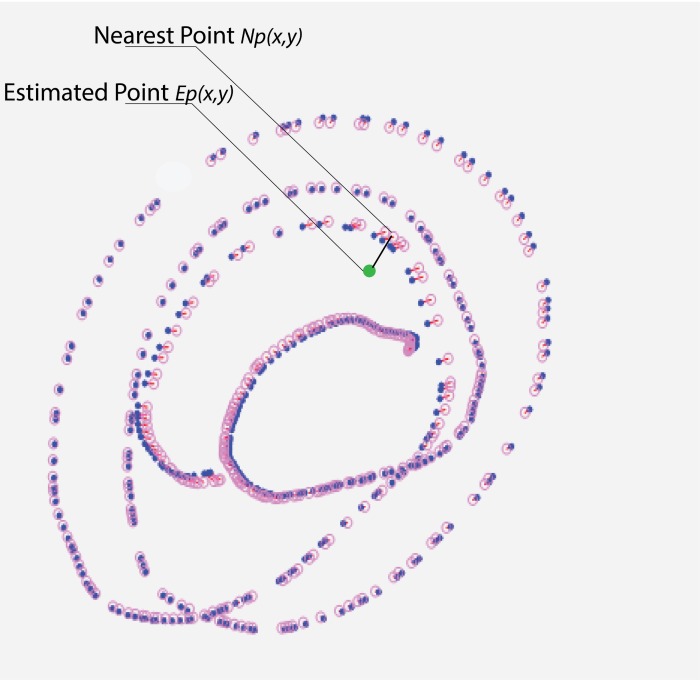
The green point is the new estimated point. From among all marker points recorded during the calibration procedure, the closest one is selected.

First, for each new estimated gaze position Ep(x, y), we search for the nearest point Np(x, y) to this point among all marker center positions gathered during the calibration procedure as shown in Figure 3.

**Figure 4:**
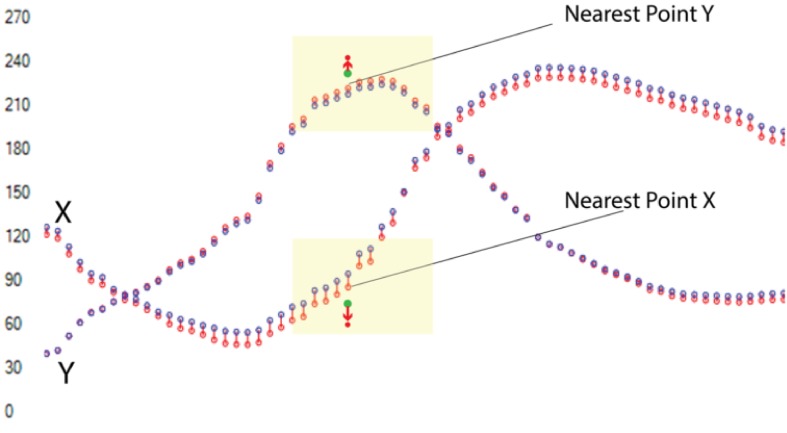
The marker points (red) and the estimated marker points (blue) are plotted for the X and Y values separately. The yellow overlays encapsulate the values to be considered for the interpolation. The upper (resp. lower) green point is the Y (resp. X) value of the interpolated point and the upper (resp. lower) filled red point is its actual position with corrected error.

Because, Ep(x, y) is estimated by the mapping function, its position is likely be incorrect with respect to its actual position. To find this potential error and correct it, we will not consider all the marker points and their relative distances as in the IDW, instead, only the marker points recorded 200 milliseconds before and after the nearest point Np(x, y) will be used. The length of the time window is adjustable and defined by the user. In this study, we chose a time window of 200 milliseconds which is large enough to encapsulate sufficient points but not too large so as to avoid introducing distant points. To properly illustrate this, Figure 4 shows the representation of the marker points and the estimated marker points for the Y and X values separately.

**Figure 5:**
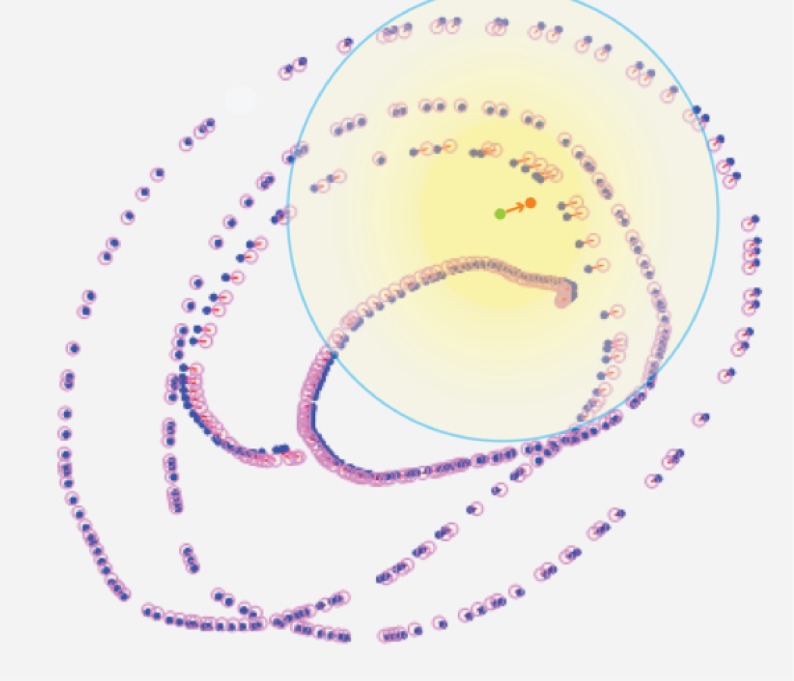
Inverse Distance Weighting impact area.

**Figure 6:**
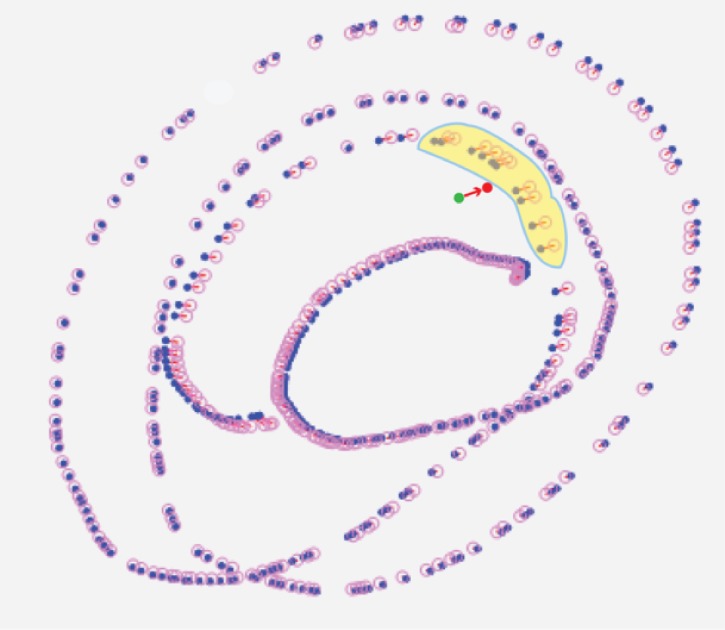
Modified Inverse Distance Weighting impact area.

The difference between the points selected using the Modified IDW method and the IDW is shown in the figures below (Figure 5 and Figure 6). The impact area using IDW is larger and outlined by a circle. The points outside the circle’s contribution are negligible or insignificant and the points inside devote more weight relative to their distance from the interpolated point. The considered points for the Modified IDW are selected as described above and represented in the yellow, blue-contoured overlay in Figure 6

## Experimental Evaluation

Through a series of calibration procedures, we investigated the results of the gaze estimations. The participants were only asked to perform the calibration procedure; all post-processing and calculations were carried out after the experiment. To this end, only data were collected during the experiment. The experiment spanned two days.

### Participants

We conducted an experiment with 12 participants, making a particular effort to include participants with different qualifications and educational levels. 3 participants were women, aged from 18 to 26 years old, and 9 were men, aged from 18 to 30 years old. The completed questionnaires showed that 2 participants were familiar with eye tracking, 2 had vaguely heard about it and 8 were completely ignorant of its existence. 6 participants were university students, 2 were researchers in human factors and 4 were Airline pilot students. Firstly, the purpose of the study was explained to the participants, thereafter they carefully read and signed a consent form. 2 participants wore glasses during the experiments.

### Apparatus and Analysis

A Pupil Labs Eye tracker was used during the experiment. The device was equipped with one world camera (sampling rate: @120Hz, resolution: 1920X1080 pixels) and one eye camera (sampling rate: @120HZ, resolution: 640X480 pixels). The computer vision algorithms used to detect and track the different targets in the world camera reduced the frame speed by 5%, and 5% of the eye camera speed was reduced by the pupil detection area and center location. Thus, pairs of points were collected at approximatively 114 frames per second for both world and eye camera.

A C# desktop software was built for the experiment including EmguCv 3.1 (an OpenCV 3.1 wrapper for C#) for the computer vision’s part implementation. The equipment setup was an XPS 15 9530 Dell Laptop 64 bits with an Intel(R) Core(TM) I7-4712HQ CPU@ 2.30GHz,2301 MHz, 4 core(s), 8 processes, 16 GB of Random Access Memory, 2GB swapping Memory. We used a 24 inches Dell 2408WFP monitor (L x W x H Dimensions: 22 x 8.17 x 15.62 inches) with a resolution of 1920x1200 pixels and a 24-millisecond response time. The marker was placed 75 cm from the participants on a plane surface (screen) to avoid introducing errors due to distortion of the target location.

### Procedure and Tasks

The study was structured as a between-subjects experiment wherein each participant of group A performed a short-time calibration procedure, and each participant of group B performed a long-time calibration procedure. We used the term short-time calibration to refer to any calibration procedure that is performed in less than 6 seconds, and long-time calibration to refer to any calibration procedure that takes more than 6 seconds. Each calibration time must not exceed 12 seconds, otherwise, it was not considered in the analysis.

First, the experimenter gave the participant general instructions and reminded him that he was going to perform a calibration procedure and that the resulting points would be stored for further processing. To avoid unintentional shifts in glances, participants were given instructions to: “Please do not speak during the calibration procedure”. The task was to look at the target and turn the head to make two rotations. Only one target was shown in the scene. In CalibMe [22], the users were asked to make a spiral pattern backward and forward with their head while fixating on the target. No such instruction was required in this study.

Once all the instructions had been given and understood, the task began. When the participant was ready to perform a calibration, he was asked to say "READY". Then the experimenter hit the button to start collecting the calibration data. At the end of the calibration, the participant was asked to remain quiet because speaking in order to inform the experimenter that the task was complete may have led to false data registration before the experimenter effectively stopped the data collection. In the same vein, if the participant hit a button on the keyboard or the mouse, false data may have been produced due to pupil shifts when looking for the key or the mouse. The participant could have been asked to leave his finger on the completion button but in this case, the participant may have focused on not losing the button during the calibration.

### Data Collection and Cleansing

The data cleansing tasks were a significant feature of the proposed study. This involved developing a cleaning process for all collected points. No filtering was done during the collection. If the calibration lasted 2 seconds, N <= 2*114 paired points were recorded as the cameras retrieved 114 frames per second. Each frame enabled the detection of one or zero points (zero if there was no detection). For each calibration, we are excluding duplicated points’ entries and their corresponding pairs.

## Results and Statistical Analysis

We are providing the results of our calibration method (in degree and in cm) from the between-subjects experiment designed with 12 participants in a laboratory. Through a series of calibrations performed by the participants, we explored the difference in results on how IDW and Modified IDW improve accuracy.

The initial analysis showed that the two methods can improve the accuracy of gaze estimation at a cost of additional time processing. We provide: • Results for raw estimated gaze data, namely the estimated points without any post processing and without correction. • Results for estimated gaze data corrected using the Inverse Distance Weighting. • And results for estimated gaze data corrected using the Modified Inverse Distance Weighting.

We found that the IDW method gave better results compared to the raw estimated data. And the Modified IDW gave the smallest Mean Angular Error (MAE) compared to the two precedents, aggregating results for all participants: • Raw Estimated Gaze data MAE: 1.644 cm (1.26°,SD=0.51°) • IDW MAE: 1.4609 cm (1.16°, SD = 0.31°) • Modified IDW MAE: 0.326 cm (0.25°,SD=0.15°).

During the experiment, we compared two calibration types: Short-time Calibration procedure (6 participants, Mean calibration Time = 4.7 seconds, SD = 0.375 seconds) and Long-time Calibration procedure (6 participants, Mean calibration Time = 9.8 seconds, SD = 0.368 seconds).

### Comparison between long & short time calibrations:

**Figure 7:**
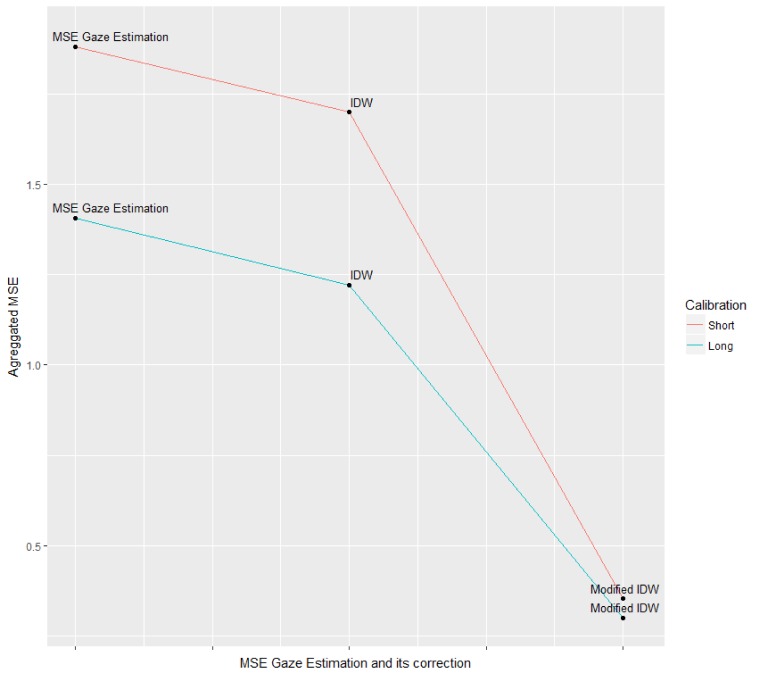
In this image, one can see that the gaze estimation’s Mean Angular Error is reduced by the Inverse Distance Weighting and is significantly reduced by the Modified Inverse Distance Weighting.

Each participant performed a calibration procedure and let’s assume Z pupil-target tuples are obtained. As in CalibMe [22], X tuples are retrieved for calibration15 points to obtain the mapping function, and the remaining Y (Z= X+Y) points are used to evaluate the gaze estimation and compute the Mean Angular Error.Figure 7 shows that the IDW helps to reduce the MAE by 0.182 cm for the Short Time Calibration procedure and 0.186 cm for the Long Time Calibration procedure. In the same vein, Figure 7 shows that error reduction is greater using the Modified IDW: by about 81.29% for the Short Time Calibration procedure (Gaze Estimation MAE = 1.882 cm vs. Modified IDW MAE =0.3524 cm) and by 78.74% for the Long Time Calibration procedure (Gaze Estimation MAE = 1.407 vs. Modified IDW MAE =0.299cm) compared to the raw estimated gaze position.

Also, compared to the standard IDW, the Modified IDW reduced the error by about 79.27% for the Short Time Calibration procedure (IDW MAE = 1.700 cm vs. Modified IDW MAE =0.3524 cm) and by 63.49% for the Long Time Calibration procedure (Gaze Estimation MAE = 1.221 vs. Modified IDW MAE =0.299cm).

### Comparison between short time calibrations:

A paired samples T-test showed that on average the Modified IDW is better than the raw gaze estimation given by the mapping function by about 1.52 cm (t = 4.777, p=0.0025) for short time calibration. Also, we found that the IDW enabled the MAE to be reduced but no significant difference between the IDW results and the raw estimated points was found statistically for short time calibration (t (1,5)= 0.95, p=0.19), however, the mean of the differences is 0.18 cm. To be concise, we are giving the mean results for the 6 participants in cm Table 1 . The extended version of this table in the Appendix gives detailed results in cm and in degree of visual angle.

**Table 1:** Mean and Standard Deviation of the results for the Short Time Calibration.

	Mean Calibration Time (sec)	No. Evaluation Points	MAE Evaluation Points (cm)	MAE Evaluation Points Corrected by IDW (cm)	MAE Evaluation Points Corrected by Modified IDW (cm)
Mean	4.7	151.667	1.88	1.700	0.352
SD	0.38	29.323	0.788	0.428	0.285

### Comparison between long time calibrations: 

The results of the comparison between long time calibrations are given in Table 2. On average the Modified IDW is statistically better than the raw estimated point results by about 1.10 cm (t = 6.04, p<0.001), based on a paired Student T-test. Although the IDW helped to reduce the MAE, no significant difference between the IDW and the raw estimated points was found statistically for long time calibration (t (1,5)= 1.7, p=0.074).

**Table 2:** Mean and Standard Deviation of the results for the Long Time Calibration procedure.

	Mean Calibration Time (sec)	No. Evaluation Points	MAE Evaluation Points (cm)	MAE Evaluation Points Corrected by IDW (cm)	MAE Evaluation Points Corrected by Modified IDW (cm)
Mean	9.83	284.833	1.407	1.221	0.299
SD	0.37	27.665	0.481	0.236	0.065

### Summary of the Calibration Assessment

Overall, the results showed that calibration time can heavily influence accuracy. When considering raw estimated points, unsurprisingly, long time calibration is most accurate. However, this is sacrificed at the cost of calibration time, as it is the lowest calibration procedure. In particular, if time is not an issue for users performing the calibration procedure, taking more time to complete the calibration has significant value in terms of accuracy for a monocular eye tracker. Nevertheless, we did not test a calibration time of more than 12 seconds. When accuracy is not an absolute need, for instance using gaze location on larger targets (big buttons, areas, etc.), one may prefer to consider a short calibration procedure. However, when using the Modified IDW method to correct raw estimated point errors, the difference between short and long calibration results is very small (0.352 cm Vs. 0.299 cm). This indicates that, instead of performing long-time calibration, approximatively the same results can be obtained with short-time calibration using the Modified IDW.

As shown, the Mean Angular Error (MAE) was usually computed to assess the quantitative evaluation of this calibration process. This corresponded to the mean of the sum of the error norms between the actual and the estimated gaze location. While this value gives a global metric to assess the quality of the calibration, in the following section, we propose visualization methods for such errors in order to offer interesting qualitative insights.

### Uncertainty and Pupil Detection

**Figure 8:**
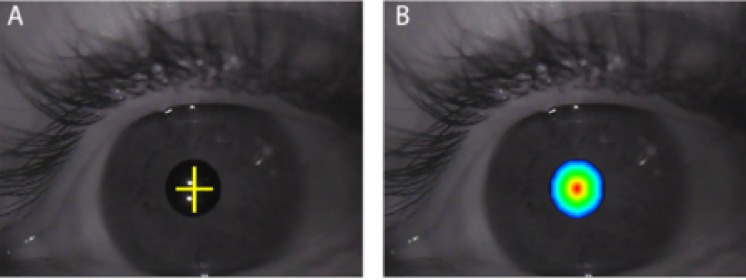
A. Pupil center position. B. Pupil location uncertainty area from 0 (Red area) to 1 (Blue area).

Understanding the eye tracking data quality is essential to the research community [12]. There are many sources of gaze estimation errors [19], including pupil dynamics [24]. Different pupil center detection algorithms exist [27, 22]. However, whatever algorithm is used, there is an inherent uncertainty over the exact position of where gaze vector “passes” through the pupil. The worst estimate is the convex polygon (or ellipse) constituting the border of the detected pupil. The pupil center position may be incorrect due to many artifacts in the eye image (experimental environment, noise, light, corneal reflection or even the algorithm itself). That is why, in this study, after the detection of the pupil center, we proposed an uncertainty area (Figure 8) from the detected center, within which the exact pupil center may be. The uncertainty varies from 0 (red color in Fig. 8(B)) to 1 (blue color in Fig. 8(B)).

### Uncertainty and the Calibration Method

The choice of calibration method is important. The mapping functions tend to give more accurate estimations in the area where the markers were placed during the calibration procedure. The common calibration method used is the nine visual stimuli arrangement in a uniform grid because it enables most parts of the calibration plan to be covered. However, whatever calibration method is used, there is still uncertainty in the gaze data processing results as the polynomial regression tends to give results that fit the calibration points and try to interpolate the points inside the calibration area. In section Calibration Correction, we proposed a method to correct the positions of the points estimated by the polynomial regression after the calibration procedure.

## Qualitative Evaluation

**Figure 9:**
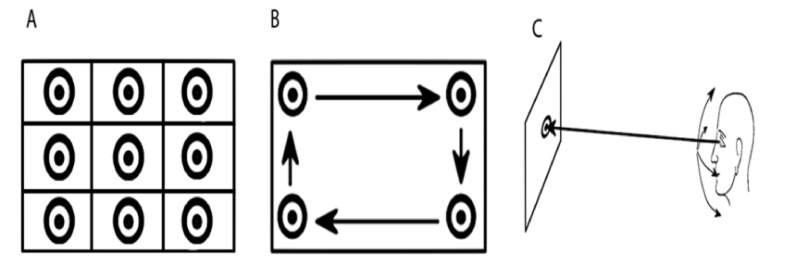
A. Common 9-point Calibration method. B. Pursuit calibration with rectangular trajectory. C. Fixed marker and head movement calibration.

Uncertainty visualization of gaze estimation during the calibration procedure provides information about the influence of the calibration setup (camera position, light conditions, calibration method) on eye tracking data quality. In this section, we illustrate the insights obtained using the visualizations generated with the proposed pipeline. By the time we had assessed and confirmed the validity of our gaze estimation method during the previous experiment, we were able to use the calibration procedure with different patterns and setups. To investigate different visualizations, we considered three different calibration procedures (Figure 9): - Classic 9-point calibration using a uniform grid, - Smooth-Pursuit calibration: where the participant is asked to fixate on a moving target on a screen. - Head rotation: where the participant is asked to rotate his head while fixating on a static target. Next, we illustrate the advantage of the proposed visualization, how it makes it possible to choose between different calibration setups, and helps with investigating the error of the calibration mapping.

### Uncertainty Visualization

**Figure 10:**
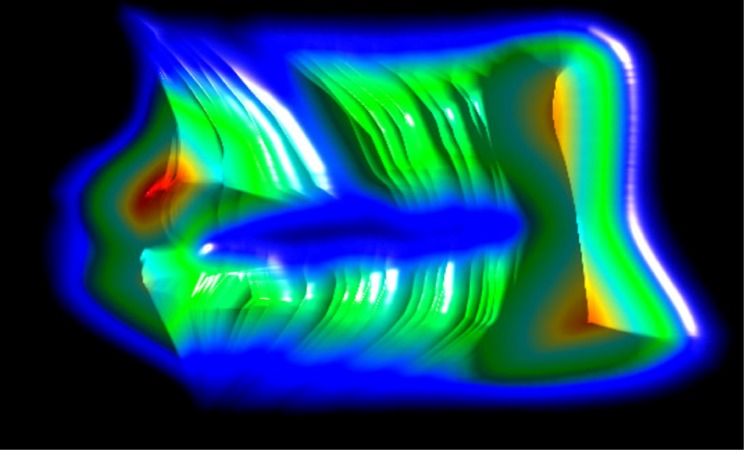
In this image, we show our uncertainty visualization results. This image corresponds to the accumulation (density map) of the recorded pupil location uncertainty. We used a bump-mapping technique to emphasize the strong variations (gradient detection). This image shows strong inaccuracy on the left part of the image and a lack of records in the center of the image.

**Figure 11:**
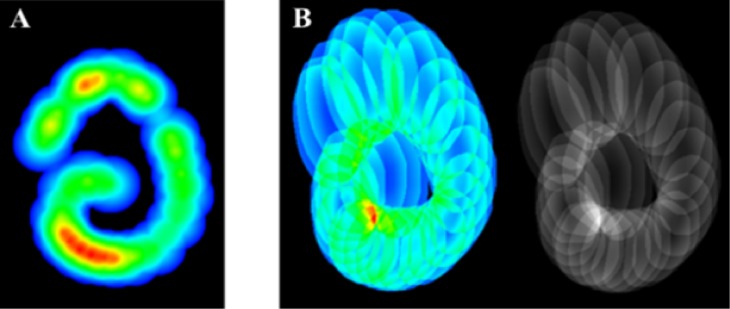
Comparison of uncertainty visualization using Gaussian kernel (A) and pupil polygon with distance transform (B) when following the circular trajectory. The right image in figure B shows the result in a different color space for more clarity.

Figure 10and Figure 11 shows the advantage of using varying shape distance transform to visualize the uncertainty of gaze estimation after calibration. Fig.11 (A) indicates only which parts of frontal field camera were covered during the calibration, and thus, corresponds to the most accurate eye data. Fig.11 (B) clearly shows that the uncertainty induced by the pupil center detection is not constant across the field camera image. In particular, we note that the ellipses are pulled vertically, indicating that the uncertainty is greater in the vertical direction compared to the horizontal. This means that the certainty of detecting a gaze shift between two objects placed on the same vertical line is higher compared to when these objects are on the same horizontal line. We also note that the ellipses are more stretched out on the upper part of the image, meaning that the uncertainty is higher when the object that a participant is looking at is placed in the upper part of the frontal field camera.

**Figure 12:**
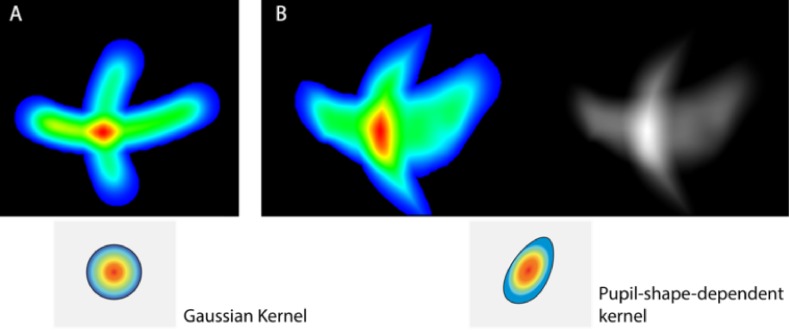
Uncertainty visualization using Gaussian kernel (A) and pupil-shape-dependent kernel (B) after performing “+” pattern calibration. The Gaussian kernel in (A) is circular, but the one used in (B) depends on the orientation, the size, and the shape of each pupil.

In addition, when following “+” alike trajectory (Figure 12), the uncertainty seems to be uniform with the Gaussian kernel, but with the varying-pupil-polygon kernel, one can clearly see that the uncertainty is greater on the right horizontal axis and tends to lessen on the lower and upper edges of the vertical axis.

### Homogeneous error

**Figure 13:**
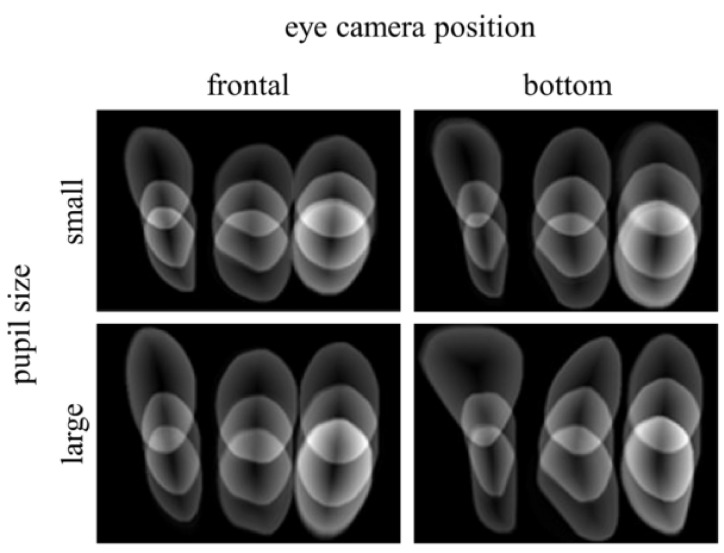
Uncertainty visualization of gaze estimation after performing 9-point calibration with two different eye camera positions (frontal and bottom) and two different pupil sizes (large and small). The size of the pupil changes with the varying lighting conditions.

Uncertainty visualization also gives us insights when comparing different eye camera positions. Figure 13 shows different visualizations corresponding to two different eye camera positions (placed in front of the eye and at the bottom) and two different pupil sizes (large and small) induced by the room lighting conditions. The images tell us that the frontal position of the eye camera is preferable because it corresponds to smaller and more homogeneous uncertainty. This is especially visible during the calibration with large pupil size. We also note that, generally, the uncertainty is higher when the calibration is performed with dimmer light conditions (and the pupil is dilated).

### Polynomial Regression Errors and Weakness

**Figure 14:**
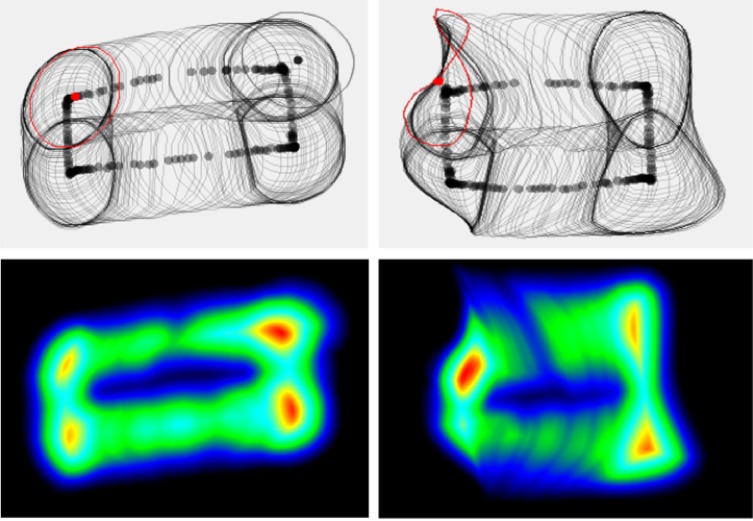
Left, pupil contours detected in the pupil camera. Right the same pupil contours process into the world camera. The outlined red contours show significant deformation due to the calibration transfer function. Bottom figures are the corresponding density maps.

The mapping function enables the interpolation of points within the calibration area during the initial stage of the calibration procedure. Figure 14 shows that the points outside the area (rectangle) result in inaccurate estimations. One can see clearly that there is a folding area on the top left corner of the estimated pupil boundary in the top right image. This folding area is due to the weakness of the polynomial regression in extrapolating points that are outside the calibration points’ area.

### Visualization of the Mapping Function Results

**Figure 15:**
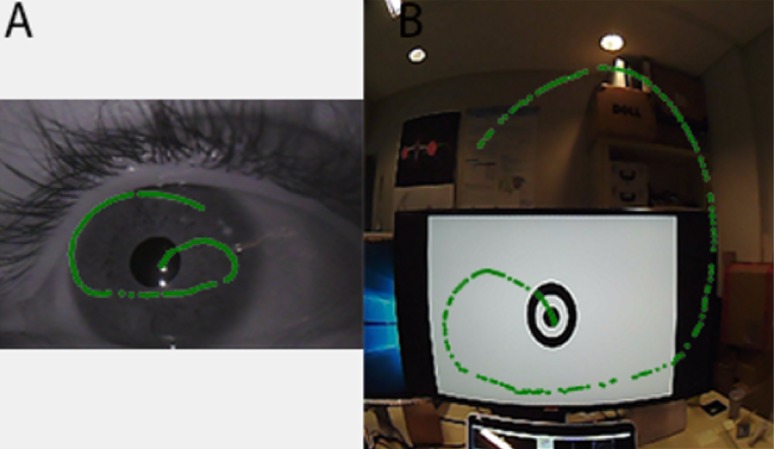
Recorded pupil location (in the pupil camera) on the left, corresponding target position on the right. Similarly, EyeRecToo proposes an innovative way to collect such calibration points using a marker on a mobile phone.

In this example, we provide evidence of visualization usages to explore the result of a calibration process in detail. As previously explained, the computation of the user gaze location is done during a calibration phase. During this calibration, a set of pupil center locations and their corresponding user gaze locations are recorded as shown in Figure 15. The calibration estimates a transfer function which turns every pupil location into its corresponding gaze location. This function is, in most feature-based calibrations, a polynomial function [1]. Considering Figure 2 A-D with recorded pupil and gaze position, one can visualize the residuals between the estimated gaze locations and their true locations. Since this error is only known where the calibration has a recorded position, we estimate the errors in every location with the Inverse Distance Weight processing [25]. In Fig. 2-D, the gaze location is corrected thanks to this estimated error.

**Figure 16:**
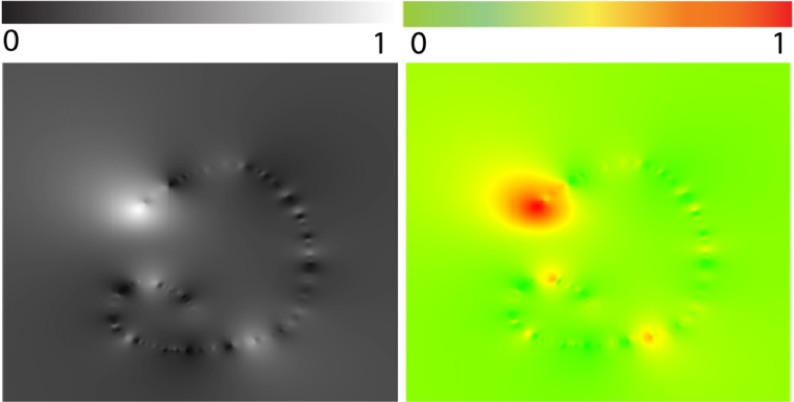
Visualization of the norm of the error between the computed gaze location and its actual position. Lower errors are dark in the left image and green in the right image.

Figure 16 shows a map of the global error estimation. While this estimation is based on the known points, one can detect that some gaze locations suffer from a high error value. In this sense, the error map provides effective insight to assess the global quality of the calibration. One possible way of improving the recorded calibration data, could be to remove the calibration points where the estimated error is too high.

## Conclusion and Further Works

In this paper, we present our gaze data visualization results to better support the understanding of calibration quality. We first explain the gaze estimation methods and through a between-subjects experiment, we showed the validity of our gaze estimation pipeline. This led us to gather calibration data and test two different methods to reduce the mean angular error. The better of the two methods yielded a mean angular error of 0.25°. Also, the experiment served to compare short and long calibration procedures and the results showed that the long calibration procedure provides better accuracy, which is in line with our thought. Next, we visually inspected the uncertainty of the whole gaze estimation pipeline taking into account, the pupil area, the mapping function, the eye camera position relative to the eye, the lighting conditions and the pupil size, which gave us an effective tool to depict the quality of the calibration.

In the near future, we plan to expand on this study in various areas. Firstly, we will provide qualitative measurements extracted from the presented visualizations. In comparison with existing measurements, which are based on data analytical computation, we will perform image-based computation and thus will qualify the visual results. Secondly, we will investigate how these produced visualizations can be a support for interaction and thus provide new interactive tools where the user can adjust the calibration process (for instance, the user may add or remove calibration points). Existing calibration systems only provide limited interaction tools and future research in this area may greatly improve calibration efficiency. We believe that this work would serve as an important guide for monocular eye tracking system calibration and uncertainty visualization.

## Appendix

Table 1a shows the extendet version of Table 1, Table 2a shows the extendet version of Table 2Table 3 shows the Calibration results for All 12 Participants

**Table 1a:** Short Time Calibration results

	Mean Calibration Time	Nb Evaluation Points	MAE Evaluation Points Cm (Degree)	MAE Evaluation Points Corrected by IDW Cm (Degree)	MAE Evaluation Points Corrected by Modified IDW Cm (Degree)
Mean	4737.167	151.666	1.88220 (1.43°)	1.70019 (1.29°)	0.35240 (0.27°)
SD	375.9912	29.3234	0.78752 (0.60 °)	0.42815 ( 0.33°)	0.28483 (0.22°)

**Table 2a:** Long Time Calibration results

	Mean Calibration Time	Nb Evaluation Points	MAE Evaluation Points Cm (Degree)	MAE Evaluation Points Corrected by IDW Cm (Degree)	MAE Evaluation Points Corrected by Modified IDW Cm (Degree)
Mean	9833.833	284.833	1.40711 (1.075°)	1.22161 (0.93°)	0.29985 (0.23°)
SD	368.413	27.6652	0.48057 ( 0.37°)	0.23570 (0.18°)	0.06472 ( 0.05°)

**Table 3:** Calibration results for All 12 Participants (Short and Long)

	Mean Calibration Time	Nb Evaluation Points	MAE Evaluation Points Cm (Degree)	MAE Evaluation Points Corrected by IDW Cm (Degree)	MAE Evaluation Points Corrected by Modified IDW Cm (Degree)
Mean	7285.5	218.25	1.64465 (1.26°)	1.46090 ( 1.16°)	0.32613 (0.25°)
SD	2685.204	74.66	0.6965 ( 0.51°)	0.4135 (0.31°)	0.1988 ( 0.15°)
